# Inclusion Body Myositis: A Case Report

**DOI:** 10.7759/cureus.81581

**Published:** 2025-04-01

**Authors:** Komalpreet Kaur, Natalie Saliba, Jitesh Kar

**Affiliations:** 1 Neurology, Edward Via College of Osteopathic Medicine, Auburn, USA; 2 Neurology, Neurology Institute of Huntsville, Inc., Huntsville, USA

**Keywords:** autoimmune, idiopathic inflammatory myopathy, inclusion body myositis, myopathy, sporadic ibm

## Abstract

Inclusion body myositis (IBM) is an autoimmune disease characterized by chronic inflammation and mainly muscular manifestations.This report details the case of a 71-year-old male presenting with gradual-onset muscle weakness in finger flexors and quadriceps, frequent falls, and muscle biopsy findings indicative of IBM. Due to a lack of curative treatments, the patient’s management focuses on supportive care, physical therapy, and mobility aids to mitigate disease progression and reduce fall risk.

## Introduction

Inclusion body myositis (IBM) is a subtype of idiopathic inflammatory myopathy, a group of disorders characterized by chronic inflammation, muscle weakness, myalgia, and reduced muscle endurance. Unlike other inflammatory myopathies, which may include multi-organ systems such as the skin, joints, lungs, heart, and gastrointestinal tract, IBM primarily affects muscle tissue with minimal extramuscular manifestations [1,2]. The clinical course of IBM can vary significantly from the other subtypes of inflammatory myopathies, influencing both the treatment course and prognosis [1]. Therefore, this study hopes to raise awareness for IBM by contributing to the current available data and further research on the topic.

There are two subtypes of IBM: sporadic and familial. Sporadic IBM is the most prevalent, primarily affecting individuals over 50 years [3,4]. The clinical phenotype is similar in both subtypes [5]. The estimated prevalence of IBM varies geographically, with an overall rate of five to nine cases per million adults. However, due to differences in diagnostic criteria and limited epidemiological data, the true prevalence remains uncertain. It is more common in men, with a male-to-female ratio of approximately 3:1 [4].

The etiology of IBM is multifactorial, involving both genetic and environmental factors. Although the exact cause remains unclear, autoimmune mechanisms play a key role. Myositis-specific autoantibodies have been identified in various idiopathic inflammatory myopathies, aiding in classification and diagnosis. IBM is particularly associated with cytosolic 5'-nucleotidase 1A (cN1A) autoantibodies [6]. The disease is characterized by an immune response involving CD8+ cytotoxic T cells and macrophages attacking muscle fibers [7]. Additionally, IBM is associated with degenerative processes, including abnormal protein aggregation and mitochondrial dysfunction, further contributing to muscle degeneration.

IBM typically presents in older adults with a gradual onset of distal and proximal muscle weakness, most notably affecting the finger flexors and quadriceps. This leads to reduced fine motor skills, difficulty walking, balance issues, frequent falls, dysphagia, and fatigue. While familial IBM exists, sporadic IBM is more common, and both share similar clinical features [5]. The diagnosis of IBM is based on clinical history, laboratory results, electrodiagnostic studies, and muscle biopsy.

This particular case highlights a patient presenting with pathognomonic clinical symptoms of forearm and quadriceps muscle weakness, distinguishing it from other forms of inflammatory myopathies that may involve additional body systems. As clinicians, it is essential to recognize this distinction through a thorough history and physical examination. Over-reliance on lab tests, biopsies, and imaging scans can lead to delayed or missed diagnoses, potentially leaving patients without answers for years. Understanding the clinical presentation of IBM is crucial because early recognition can prevent unnecessary testing, expedite diagnosis, and improve patient outcomes. Learning about IBM helps clinicians provide more targeted care and avoid the frustration of misdiagnosis, ultimately ensuring better management of this progressive condition.

## Case presentation

A 71-year-old male with a six-year history of gradual, painless weakness in both lower extremities and hands presented with difficulty walking, standing up from a seated position, and frequent spontaneous falls. He also reported difficulty opening bottles and reduced manual dexterity. He denied any clear inducing factors or preceding illnesses. The patient also denied problems with chewing, swallowing, breathing, bladder function, or bowel function. The patient had no known family history of neuromuscular disease, though his maternal grandfather had a history of "polyneuritis," leading to bedridden status in his final years.

On physical examination, the patient exhibited significant muscle atrophy in the thighs and moderate atrophy in the forearms, more pronounced on the right side. Knee and ankle reflexes were diminished, but biceps, triceps, and brachioradialis reflexes were 2+ bilaterally. Muscle weakness was noted in multiple muscle groups, including wrist flexors, finger flexors, and quadriceps, with preserved sensation in all extremities. The full neurological exam findings are listed in Table 1.

**Table 1:** Neurological examination findings Reflexes are graded on a scale from 0 to 4+, where 0 = absent, 1+ = hypoactive, 2+ = normal, 3+ = hyperactive, and 4+ = clonus. Muscle strength is graded on a 0-5 scale, where 0 = no movement, 1 = trace of muscle contraction but no movement, 2 = movement possible but not against gravity, 3 = movement possible against gravity but not against resistance, 4 = movement against some resistance but weaker than normal, 5 = full movement against full resistance. Plus sign (+) next to a muscle strength indicates slightly stronger strength than the current grade, but still below the next grade scale.

Category	Subcategory	Right	Left
Mental status	Concentration	Normal	
	Attention	Normal	
	Language	Normal	
	Speech	Normal	
Cranial nerves	II-XII normal bilaterally		
Tone and trophic state	Normal tone throughout		
	Marked muscle atrophy in thighs, worse on right		
	Moderate muscle atrophy in forearms, worse on right		
Reflexes	Biceps reflex	2+	2+
	Triceps reflex	2+	2+
	Brachioradialis reflex	2+	2+
	Knee reflex	0	0
	Ankle reflex	0	0
Motor strength	Neck flexion	5	5
	Neck extension	5	5
	Shoulder flexion	5	5
	Shoulder extension	5	5
	Shoulder abduction	5	5
	Shoulder adduction	5	5
	Elbow flexion	5	5
	Elbow extension	5	5
	Wrist flexion	4+	4+
	Wrist extension	5	5
	Finger abduction	4+	4+
	Finger adduction	4	4+
	Finger flexion	4+	4+
	Finger extension	4	4
	Hip flexion	4	4
	Hip extension	5	5
	Hip abduction	5	5
	Hip adduction	5	5
	Knee flexion	4+	4+
	Knee extension	3+	4+
	Ankle dorsiflexion	2	3+
	Ankle plantarflexion	5	5
	Toe flexion	4	4+
	Toe extension	4	4+
Sensory exam	Light touch	Normal in upper and lower extremities bilaterally	
	Pinprick	Normal in upper and lower extremities bilaterally	
Gait	Not tested (in wheelchair)		
Coordination	Finger-to-nose	Normal with superimposed action tremor	
	Rapid alternating movements	Normal with superimposed action tremor	
	Heel-to-shin	Normal	

Electrodiagnostic study revealed chronic, moderately severe, partially irritable myopathy involving the right anterior thigh compartment and right forearm flexors and extensors. There was also evidence of superimposed chronic, mildly severe L4-S2 and C8-T1 radiculopathies on the right side.

MRI scan of cervical spine revealed mild degenerative spondylosis with disk bulges at C4-C5, C5-C6, and C6-C7 levels with mild central canal stenosis. A muscle biopsy of the left vastus lateralis indicated IBM, and it also demonstrated features of denervation atrophy and partial reinnervation.

The patient was advised to continue physical therapy to maintain muscle function while avoiding excessive exertion that could result in muscle injury and accelerate muscle degeneration. Due to the progressive nature of IBM and fall risk, the use of a wheelchair and ambulation aids, such as a cane or rollator, was recommended.

## Discussion

IBM is a slowly progressive autoimmune disorder with an insidious onset and asymmetric muscle involvement. It predominantly affects distal muscles, particularly the finger flexors, and proximal lower extremity muscles, such as the quadriceps. This distinctive pattern helps to differentiate IBM from other myopathies. Patients commonly present with frequent falls due to quadriceps weakness, and more than 50% experience difficulty swallowing (dysphagia) due to pharyngeal muscle involvement [2].

The pathogenesis of IBM involves an autoimmune-mediated attack on muscle fibers by CD8+ T cells and macrophages. These immune cells infiltrate muscle tissue, leading to chronic inflammation and muscle damage. Upregulation of major histocompatibility complex (MHC) class I antigen on muscle fibers is another hallmark feature, contributing to the immune response [7]. The presence of anti-cN1A autoantibodies further supports the autoimmune basis of the disease [6,8]. Additionally, IBM is associated with degenerative mechanisms, including protein misfolding and mitochondrial abnormalities [9]. These processes contribute to the progressive nature of the disease and resistance to immunosuppressive therapy.

**Figure 1:**
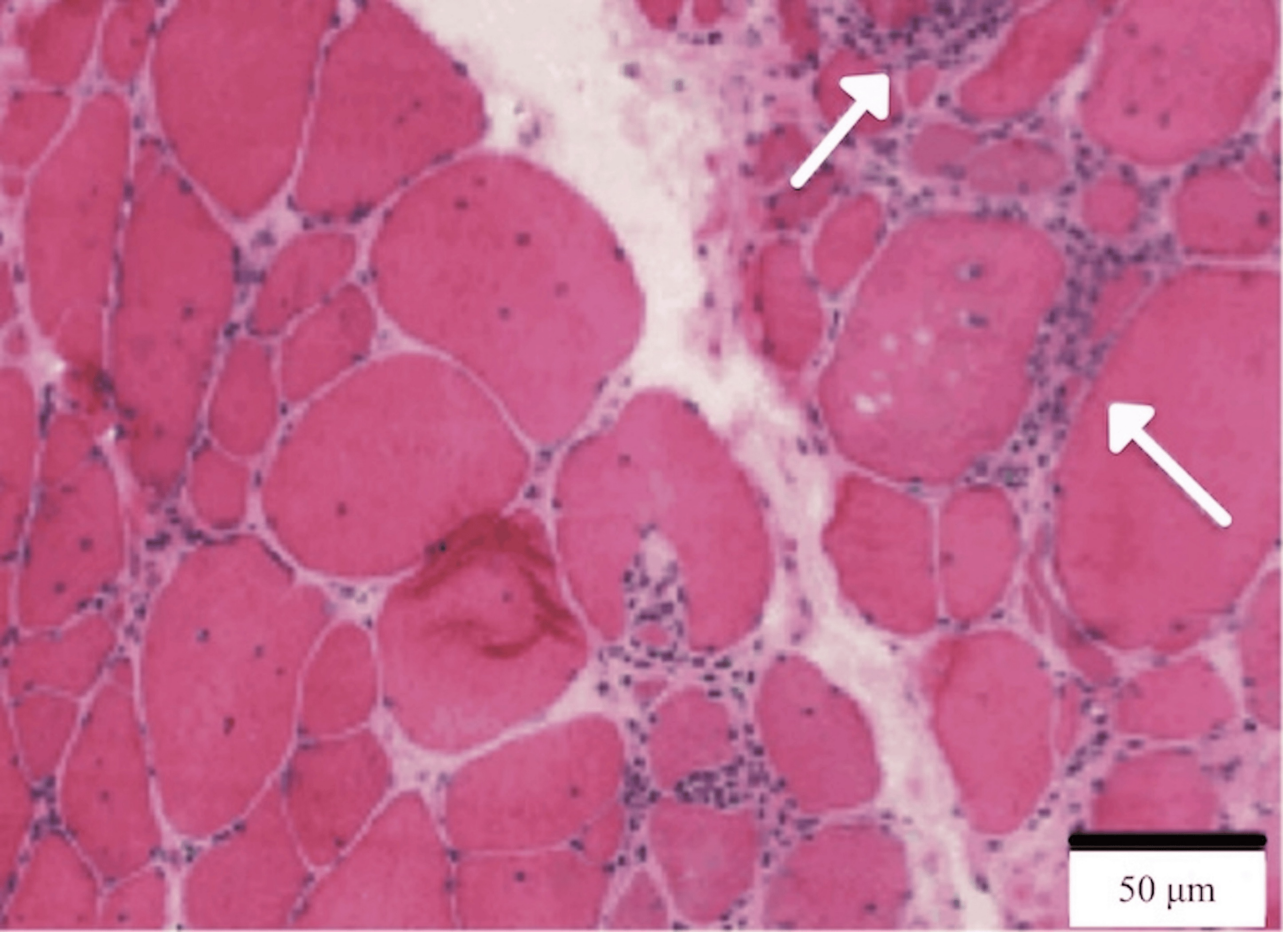
Histological muscle biopsy of a patient with sporadic IBM. White arrows indicate the endomysial inflammatory reaction, while a global dystrophic pattern is observed. The muscle biopsy was stained with hematoxylin and eosin, which stains nuclei blue and muscle fibers pink. This image was captured at 200x magnification. Source: Ref. [9].

**Figure 2:**
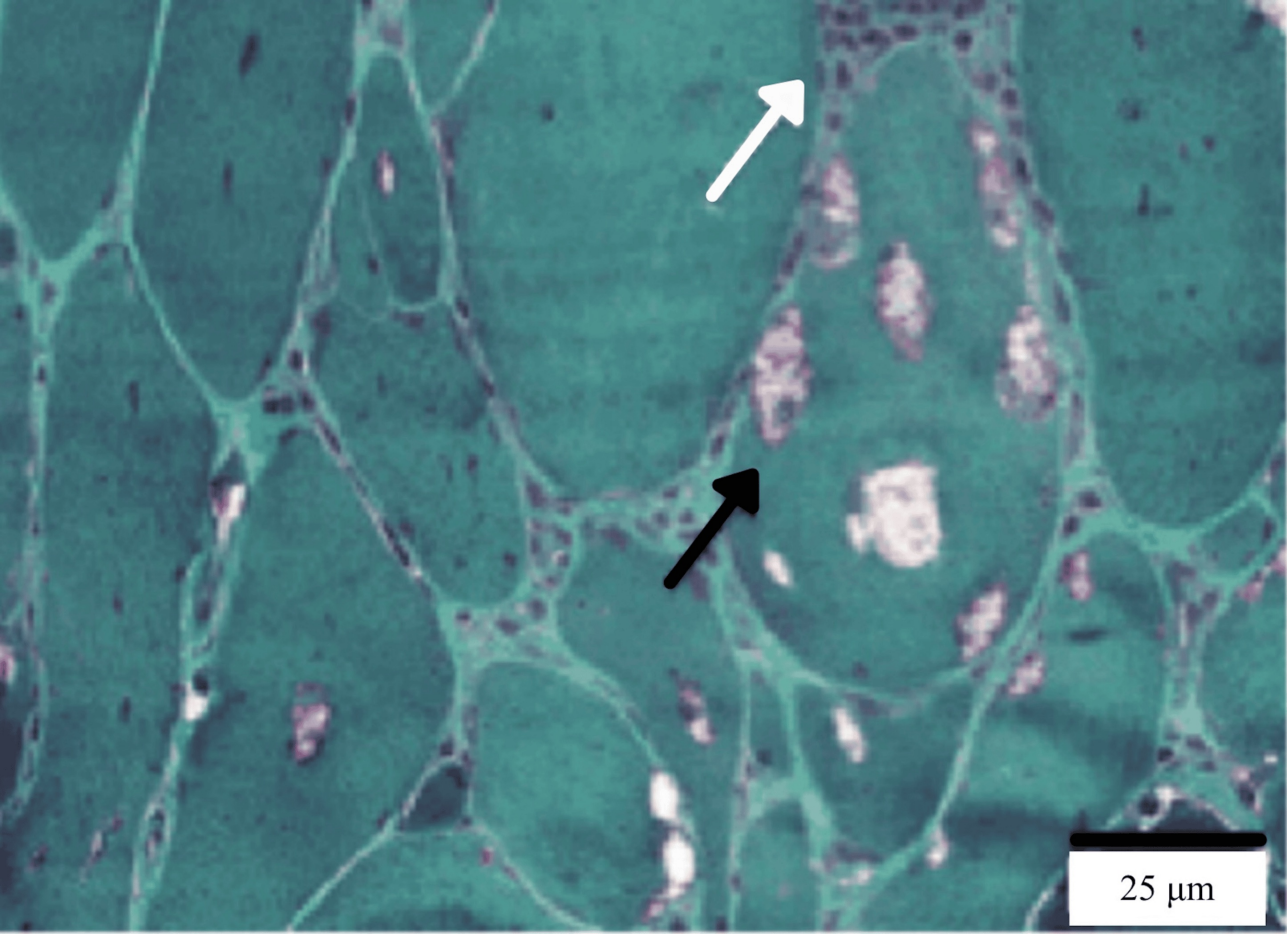
Histological muscle biopsy of a patient with sporadic IBM. White arrow highlights the endomysial inflammatory reaction, while the black arrow indicates rimmed vacuoles. The muscle biopsy was stained with Gomori’s trichrome, which stains muscle fibers and cytoplasm in red, nuclei in black, and collagen-containing connective tissues in blue. This image was captured at 400x magnification. Source: Ref. [9].

Diagnosis relies on a combination of clinical features, laboratory tests, electrodiagnostic studies, and muscle biopsy. Hallmark clinical findings include progressive muscle weakness lasting over 12 months, onset after age 45, and predominant involvement of distal muscles in the upper limbs and proximal muscles in the lower limbs. Laboratory findings often reveal mildly elevated creatine kinase levels (<15 times the upper limit of normal) and the presence of anti-cN1A autoantibodies [8]. Electrodiagnostic studies may show a combination of myopathic and neurogenic changes [3]. The muscle biopsy findings confirm the diagnosis of IBM and exhibit its characteristic features. Endomysial inflammatory infiltrates, where immune cells invade the muscle tissue within the connective tissue (endomysium) surrounding individual muscle fibers, are observed, as shown in Figures 1, 2 with white arrows.

Additionally, there is an upregulation of MHC class I proteins, which are found on the surface of muscle cells, signaling the immune system to attack and contributing to muscle damage. Rimmed vacuoles, appearing as small bubble-like spaces within muscle fibers, indicate cellular breakdown, as shown in Figure 2 by the black arrow. Abnormal protein accumulation occurs due to the presence of misfolded proteins that muscle cells cannot clear, leading to dysfunction. Furthermore, tubulofilamentous inclusions, which are 15-18 nm filamentous structures seen on electron microscopy, serve as a hallmark of IBM [5,8,9].

IBM remains a challenging disease to treat, with current therapies primarily focused on supportive care. Initially, the patient used a cane for support but later transitioned to a rollator as his condition progressed. Due to frequent falls from worsening muscle tone, he now relies on a wheelchair for most of his mobility. He has also noticed an increasing frequency of episodes where his muscles suddenly give way, which he describes as "muscle collapse." These unpredictable episodes, often triggered by standing up after sitting for prolonged periods, result in sudden, spontaneous losses of strength and frequent falls. Conservative management helps maintain function and improve daily living activities for inflammatory myopathy [10]. Balance therapy is crucial to minimize fall risk. The patient participates in physical therapy twice a year for a few weeks at a time and continues the exercises at home. Additionally, the patient engages in aquatherapy, as the buoyancy of water allows him to perform movements that would be difficult on land. Dysphagia may be managed with dietary modifications or swallowing therapy. However, the patient denies difficulty chewing or swallowing. In severe cases, intravenous immunoglobulin (IVIG) or surgical interventions may be considered [4,11]. Interestingly, it is important to highlight the difficulty individuals face in coming to terms with a diagnosis that is both incurable and imposes significant physical limitations. For the patient’s psychological well-being, he has joined a Facebook support group, which helps him cope with the challenges of his condition by connecting with others facing similar struggles.

Given the complexity of IBM, it is crucial to distinguish it from other inflammatory myopathies, such as polymyositis and dermatomyositis, as these diagnoses involve different prognoses and management strategies. Unlike polymyositis and dermatomyositis, which often present with more generalized proximal muscle weakness and may involve extramuscular manifestations (such as skin changes in dermatomyositis), IBM typically affects the distal muscles, particularly the forearms and quadriceps. The progressive, asymmetric muscle weakness seen in IBM, often with specific involvement of the finger flexors and quadriceps, is a hallmark distinguishing feature [1,2]. In this case, the patient exhibits the classic presentation of IBM, with the characteristic pattern of distal muscle weakness, particularly in the forearms and quadriceps, alongside a gradual, progressive course. This distinction is critical, as over-reliance on lab tests, biopsies, or imaging could delay diagnosis and management, underscoring the importance of clinical judgment in differentiating these conditions based on a thorough history and physical examination.

Although no definitive treatment exists, some immunosuppressive therapies, including glucocorticoids, methotrexate, cyclophosphamide, azathioprine, and IVIG, have been used with limited success. Ongoing clinical trials exploring monoclonal antibodies and interleukin receptor blockers show promise for future therapeutic advancements [4].

IBM is a progressive disease with an unchanged life expectancy, but complications such as aspiration pneumonia from dysphagia, respiratory muscle weakness, and fall-related injuries can reduce survival [12,13].

## Conclusions

IBM is a chronic, progressive myopathy that predominantly affects individuals above 50 years old. It is characterized by distal upper limb and proximal lower limb muscle weakness, forearm and thigh muscle atrophy, and minimal extramuscular involvement. Diagnosis is confirmed by clinical features, laboratory testing, electrodiagnostic studies, and muscle biopsy. While no curative treatment exists, supportive care, including physical therapy and assistive devices, is key to maintaining function and reducing complications. Emerging therapies offer hope for future disease-modifying treatments.

Despite IBM's recognition as a distinct clinical entity, limited epidemiological data restrict a comprehensive understanding of its true prevalence and natural history. Variations in geographic differences and underdiagnosis contribute to uncertainty in IBM’s incidence. Expanding research efforts are essential for gaining a clearer picture of disease burden and developing effective treatments. Emerging therapies, including monoclonal antibodies and immune modulators, offer hope for future disease-modifying treatments.
